# Genetic and epigenetic loss of microRNA-31 leads to feed-forward expression of EZH2 in melanoma

**DOI:** 10.18632/oncotarget.622

**Published:** 2012-08-31

**Authors:** Irfan A. Asangani, Paul W. Harms, Lois Dodson, Mithil Pandhi, Lakshmi P. Kunju, Christopher A. Maher, Douglas R. Fullen, Timothy M. Johnson, Thomas J. Giordano, Nallasivam Palanisamy, Arul M. Chinnaiyan

**Affiliations:** ^1^ Michigan Center for Translational Pathology; ^2^ Department of Pathology, University of Michigan; ^3^ Department of Dermatology, University of Michigan; ^4^ Center for Computational Medicine and Bioinformatics; ^5^ Comprehensive Cancer Center, University of Michigan Medical School; ^6^ Howard Hughes Medical Institute, University of Michigan Medical School; ^7^ Department of Urology, University of Michigan; ^8^ Shared senior authors

**Keywords:** microRNA-31, melanoma, tumor suppressor, EZH2, DZNep

## Abstract

MicroRNAs (miRs) play a key role in cancer etiology by coordinately repressing numerous target genes involved in cell proliferation, migration and invasion. The genomic region in chromosome 9p21 that encompasses miR-31 is frequently deleted in solid cancers including melanoma; however the expression and functional role of miR-31 has not been previously studied in melanoma. Here, we queried the expression status and performed functional characterization of miR-31 in melanoma tissues and cell lines. We found that down-regulation of miR-31 was a common event in melanoma tumors and cell lines and was associated with genomic loss in a subset of samples. Down-regulation of miR-31 gene expression was also a result of epigenetic silencing by DNA methylation, and *via* EZH2-mediated histone methylation. Ectopic overexpression of miR-31 in various melanoma cell lines inhibited cell migration and invasion. miR-31 targets include oncogenic kinases such as SRC, MET, NIK (MAP3K14) and the melanoma specific oncogene RAB27a. Furthermore, miR-31 overexpression resulted in down-regulation of EZH2 and a de-repression of its target gene rap1GAP; increased expression of EZH2 was associated with melanoma progression and overall patient survival. Taken together, our study supports a tumor suppressor role for miR-31 in melanoma and identifies novel therapeutic targets.

## INTRODUCTION

Melanoma is the fifth most common cancer in the United States, with nearly 70,000 new diagnoses and 9000 deaths annually [1]. Localized disease is managed by simple excision however advanced metastatic disease often requires chemotherapy, immune therapy, and/or small molecule inhibitors of BRAF, none of which are curative in most patients. Therefore, prognosis for advanced melanoma remains very poor [2].

Melanomas are genetically complex malignancies characterized by dysregulation of multiple signaling and tumor suppressor/oncogene pathways, including *BRAF*, *NRAS*, *PTEN*, and/or *CDKN2A [3-5]*. microRNAs (miRs) play an important role in multiple cancers including melanoma, and may act either as oncomiRs by targeting tumor suppressors, or as tumor-suppressive miRs by targeting oncogenes [6].

miR-31 is emerging as a complex player in a number of cancers. Evidence suggests miR-31 can acts as either an oncomiR or a tumor-suppressive miR in a tumor type-specific manner. As an oncomiR, miR-31 is overexpressed and promotes proliferation and/or invasion in carcinoma of the lung, colon, and head and neck [7-13], whereas it plays a tumor-suppressive role in regulating proliferation, invasion, and metastasis in other tumor types [13-18]. The parent gene for miR-31, *hsa-mir-31*, is located on 9p21.3, a chromosomal region frequently deleted in melanoma and other malignancies [18-20]. However, investigations of miR-31 expression and function in melanoma have been limited and not much is known about miR-31 target genes in melanoma.

EZH2, the catalytic subunit of Polycomb Repressive Complex 2 (PRC2) is overexpressed in various solid cancers and is associated with metastatic progression and poor prognosis [21-23]. EZH2 is a transcriptional repressor that catalyzes histone H3K27 trimethylation [24] and its upregulation in cancer is due in part to MEK-ERK-mediated transcriptional activation and/or by loss of miR-101 that negatively regulates EZH2 expression by binding to its 3'-UTR [25-28]. However, the expression status and mechanisms of EZH2 overexpression are also poorly understood in melanoma.

In this study, we characterized the expression status and functional role of miR-31 and validated several of its important target genes in melanoma. We found recurrent genomic loss of miR-31 and abrogated expression by epigenetic silencing through DNA methylation and EZH2-mediated histone H3K27 trimethylation. Transient overexpression of miR-31 in various melanoma cell lines inhibited cell migration and invasion, supporting a role for miR-31 as a tumor-suppressive miR that targets multiple oncogenes such as SRC, MET, NIK and RAB27a. Finally, we found that miR-31 negatively regulates the expression of EZH2 that may in turn mediate epigenetic silencing of miR-31 resulting in a mutually antagonistic feedback loop.

## RESULTS

### Recurrent deletion of miR-31 locus in melanoma

**Figure 1:**
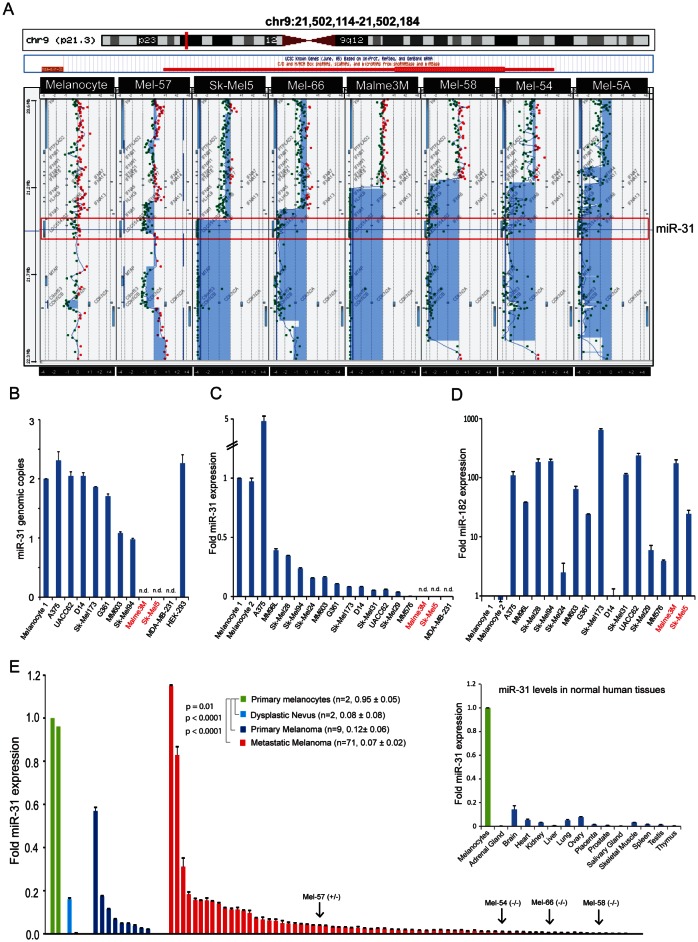
miR-31 displays decreased expression in melanoma, and is deleted in a subset of melanoma tumors A, genomic aberrations at 9p21 (indicated by the red vertical line on the chromosome schematic in top panel) were examined by array comparative genomic hybridization, with the green and red dots indicating genomic location of the probes used in the microarray. The *hsa-miR-31* locus is highlighted by a red box. B, genomic status of miR-31 in melanoma cell lines by qPCR of genomic DNA. The breast carcinoma cell line MDA-MB-231 intended to be a control displays homozygous deletion of miR-31. Normal melanocytes and HEK-293 genomic DNA served as control. C, miR-31 expression in normal melanocytes and melanoma cell lines as determined by qRT-PCR. D, expression of the oncogenic microRNA miR-182 in melanoma cell lines relative to melanocytes. E, miR-31 expression in dysplastic nevi tested (light blue), primary melanoma (dark blue) and metastatic melanoma (red) relative to benign melanocytes (green). Average fold miR31 expression and standard error of the mean for each group are shown. P-values are relative to benign melanocytes. (*Inset*) miR-31 expression in melanocytes (green) relative to other benign human tissues (blue).

In order to better understand the genomic aberrations of melanoma at the copy number level, we performed array–comparative genomic hybridization (aCGH) analysis of twenty-six melanoma tissue samples and fifteen melanoma cell lines along with primary melanocytes as control. We focused primarily on the genomic region of chromosome 9p21 since this region containing *cdkn2a* and *cdkn2b* is known to be deleted in multiple solid and hematologic malignancies [29]. We found deletion in *cdkn2a* and *cdkn2b* loci in 50% of the samples analyzed (21/42) that included both focal and larger homozygous and heterozygous deletion. Furthermore, a subset of samples (9/42, 21%) displayed larger deletions of chromosome 9p that includes the region encoding miR-31 (Figure 1A). Out of fifteen melanoma cell lines analyzed by aCGH, Malme3M and Sk-Mel5 displayed complete loss of miR-31 locus, whereas primary melanocytes did not harbor any deletions.

### Loss of lineage-specific miR-31 expression in primary and metastatic melanoma

Following the aCGH analysis in melanoma tissues and cell lines, we further validated the allelic imbalance of miR-31 by genomic PCR. As expected, no PCR-amplified product was observed in Malme3M and Sk-Mel5 cell lines that harbor homozygous deletion of miR-31 locus as determined by aCGH (Figure 1B). Furthermore, interphase fluorescence *in situ* hybridization (FISH) analysis of Malme3M showed a clear loss of miR-31 genomic region (Supplementary Figure 1). Analysis of two other cell lines MM603 and Sk-Mel94 revealed a one copy loss of miR-31; however, five additional melanoma cell lines retained normal copy numbers. Furthermore, we serendipitously found that the MDA-MB-231 breast cancer cell line that was shown to lack miR-31 expression [30] has a homozygous deletion of miR-31 locus (Figure 1B), while HEK-293 cells and melanocyte controls had normal copies. Additionally, mature miR-31 expression levels were evaluated in a panel of 15 melanoma cell lines and two primary melanocytes with MDA-MB-231 cell line serving as control. As depicted in Figure 1C, all melanoma cell lines except A375 exhibited lower levels of miR-31 compared to primary melanocytes, with Sk-Mel5, Malme3M and MDA-MB-231 showing no signal for mature miR-31. As a control, miR-182, an oncogenic miR in melanoma [31], was significantly overexpressed in all the melanoma cell lines compared to normal melanocytes, confirming the robustness and sensitivity of our PCR-based miR detection (Figure 1D).

miR-31 was reported as both an oncogene and a tumor suppressor gene based on its expression status in various cancers. To determine the expression level of miR-31 in melanoma tissues, we analyzed dysplastic nevus (n=2), primary melanoma (n=9) and metastatic melanoma (n=71) samples for transcript levels of mature miR-31 by PCR quantification. miR-31 expression levels in dysplastic nevus, primary and metastatic melanoma samples were significantly decreased compared to primary melanocytes (Figure 1E). Importantly, one metastatic melanoma sample harboring heterozygous loss of miR-31 (Mel-57) and three of the samples with homozygous deletion (Mel-54, 58 and 66) of miR-31 (Figure 1A) displayed remarkable concordance with miR-31 transcript levels. The residual miR-31 transcripts detected in these samples is likely due to normal stromal contamination of the samples. In addition, among various normal tissues, melanocytes displayed the highest miR-31 expression (Figure 1E inset). These data indicate that melanocytes are generally enriched with miR-31, and during the progression towards melanoma, miR-31 expression is abrogated either by genomic loss or by epigenetic silencing.

### DNA demethylation as well as EZH2 depletion by siRNA or DZNep treatment induces miR-31 expression

**Figure 2:**
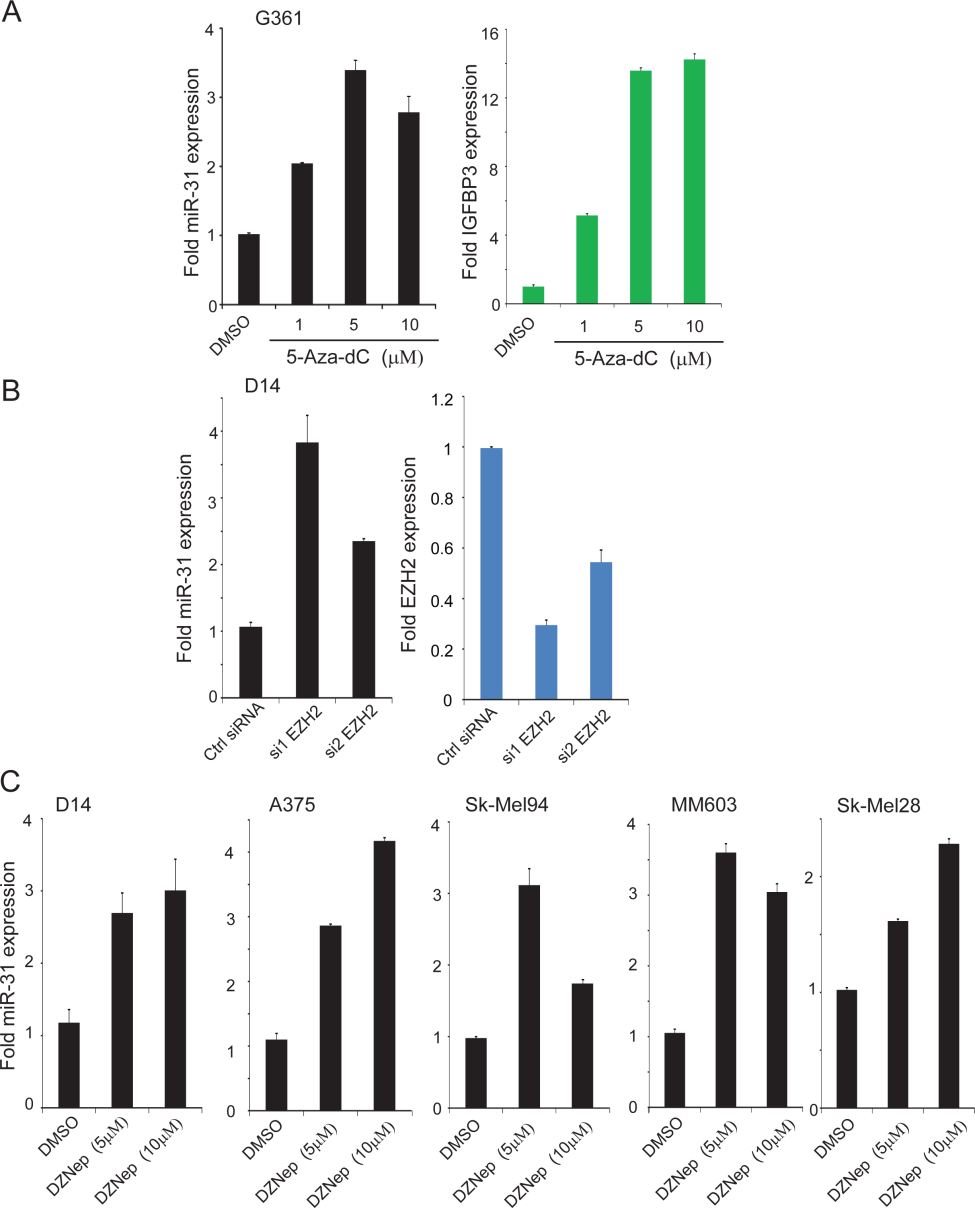
miR-31 expression is downregulated by epigenetic silencing in melanoma A, qRT-PCR for miR-31 using total RNA extracted from G361 treated with 5'-aza-dC melanoma cells. IGFBP3 (green) is included as a positive control for drug treatment. B, Expression of EZH2 (blue) and miR-31 (black) upon EZH2 knockdown in D14 melanoma cells. C, expression of miR-31 after treatment with global histone methylation inhibitor deazaneplanocin A (DZNep) in various melanoma cell lines.

A detailed query of miR-31 map from the UCSC genome browser revealed that the gene is encoded in an intron of MIR31HG (miR-31 host gene), a 105kB non-coding gene containing four exons (Supplementary Figure 2) located on chromosome 9p21.3. The transcriptional regulatory region of MIR31HG was enriched for the H3K27Ac mark (ENCODE ChIP-seq data) that is associated with transcriptionally active genes. Furthermore, this regulatory region consists of 77 CpG islands surrounding the transcription start site (TSS). ENCODE ChIP-seq tracks of NT2-D1 pluripotent human testicular embryonal carcinoma cell line were enriched for H3K27me3 transcriptional silencing mark in its regulatory region (Supplementary Figure 2). This observation suggests that miR-31 transcription could be silenced by CpG island methylation or by histone H3K27me3. Indeed, transcriptional silencing by CpG island methylation and H3K27me3 mediated by EZH2 have been reported in breast cancer and ATL leukemia [32, 33]. In order to test whether miR-31 locus is epigenetically silenced CpG island methylation of its regulatory region in melanoma, we treated G361 cells that have normal miR-31 copy number (Figure 1B) with a DNA methylation inhibitor, 5'-aza-2'deoxyCytidine (5-Aza-dC). The IGFBP3 gene, known to be hypermethylated in melanoma and other cancers [31, 34], was used as a positive control and was highly expressed upon 5-aza-dC treatment (Figure 2A, right panel). More importantly, miR-31 expression was also induced upon 5-aza-dC treatment (Figure 2A left panel) indicating that miR-31 transcription is silenced by DNA methylation in melanoma cells. Moreover, miR-31 was shown to be repressed by EZH2 through H3K27me3 in other cancer types [33, 35].

We next determined whether EZH2 regulates miR-31 expression in melanoma cells. D14 cells were transfected with two independently validated EZH2 siRNA (Figure 2B right panel) and expression levels of miR-31 were measured. The levels of miR-31 were markedly increased upon EZH2 knockdown (Figure 2B left panel). Next, treatment of five different melanoma cell lines with deazaneplanocin A (DZNep), a compound that, which depletes EZH2 and other polycomb repressive complex 2 (PRC2) components and thus attenuates H3K27me3 [36], led to the induction of miR-31 in all cell lines (Figure 2C). These results support the notion that miR-31 is epigenetically silenced by DNA methylation and EZH2-mediated histone methylation in melanomas.

### miR-31 inhibits migration/invasion of melanoma cells

**Figure 3:**
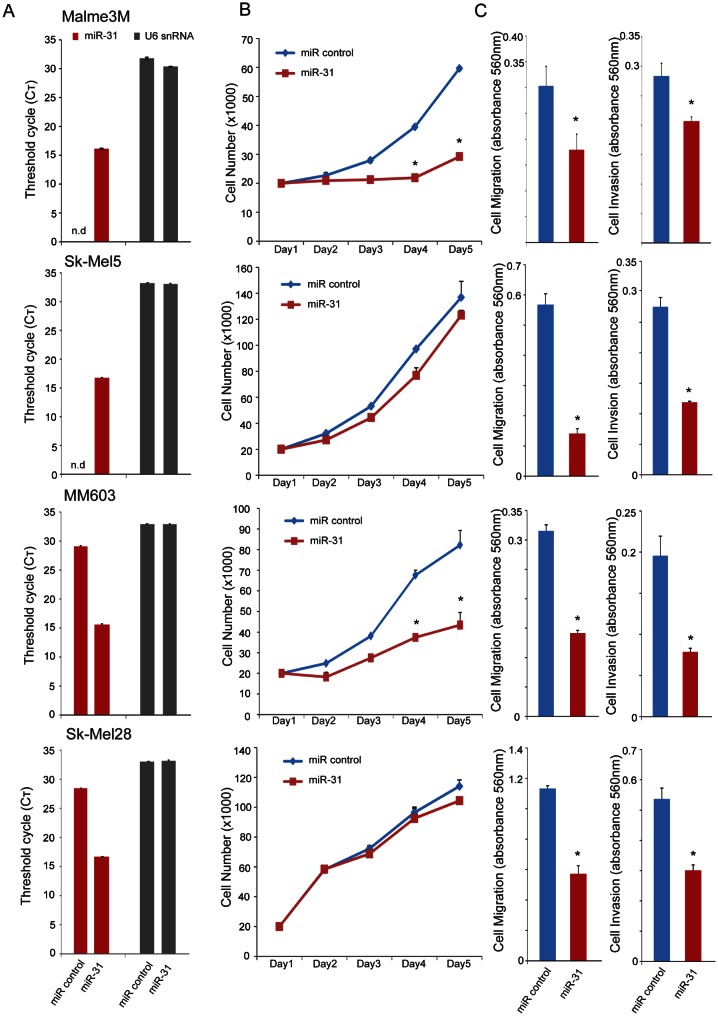
Effect of miR-31 overexpression on proliferation, migration, and invasion of melanoma cells A, qRT-PCR for miR-31 using total RNA from various melanoma cell lines after transient transfection of precursor miR-31 (pre-miR-31) or control miR. U6 snRNA serves as an endogenous control. Raw Ct values are plotted for both miR-31 and U6 snRNA. B, effect of miR-31 overexpression on proliferation in melanoma cell lines. Cells were quantitated by Coulter Counter at the indicated time points to monitor growth. C, effect of miR-31 overexpression on migration through Transwell and invasion through Matrigel in melanoma cell lines. Migrated and invaded cells were stained with crystal violet, solubilized and quantitated by measuring absorbance at 560 nm.* indicates p<0.05, Student's *t*-test.

We next examined the functional role of miR-31 in context of oncogenic phenotypes such as cell proliferation, migration and invasion of melanoma cell lines. Transient overexpression of miR-31 in Malme3M and Sk-Mel5 cells (Figure 3A) harboring genomic deletion of miR-31 resulted in divergent phenotypes for cell proliferation; Malme3M cell growth was significantly inhibited whereas Sk-Mel5 cells were unaffected (Figure 3B). However, both cell lines displayed significant reduction in migration and invasion upon miR-31 overexpression as measured by modified Boyden chamber assays (Figure 3C). Likewise, MM603 and Sk-Mel28 cells that have at least one normal copy of miR-31 also exhibited differential effect in cell proliferation upon miR-31 overexpression, with MM603 cell growth significantly inhibited while Sk-Mel28 cells were unaffected. Again, both these cell lines showed marked inhibition in their migration and invasion potential upon miR-31 overexpression. A375 cells that express high levels of endogenous miR-31 (Figure 1C) displayed proliferation advantage in the serum-free condition upon miR-31 knockdown (Supplementary Figure 3). Overall, these data suggest that miR-31 is involved in cell migration and invasion in melanoma similar to its role in breast cancer [30].

### miR-31 targets include melanoma oncogenes SRC, NIK, RAB27a and MET

**Figure 4:**
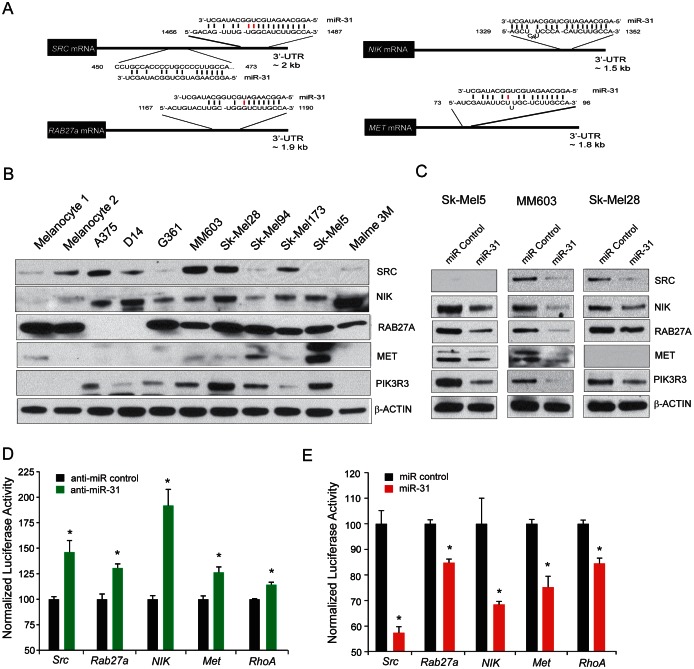
Targets of miR-31 in melanoma A, schematic of predicted miR-31 binding sites in 3' UTR of putative miR-31 targets, with complementary pairs shown in black and mismatches shown in red. B, immunoblot analysis of predicted miR-31 targets SRC, NIK, RAB27a, and MET in melanoma cell lines and benign melanocytes. PIK3R3, an indirect target of miR-31, is also included. β-Actin was used as loading control. C, immunoblot analysis of predicted miR-31 target proteins SRC, NIK, RAB27A, and MET, upon miR-31 overexpression. PIK3R3 as an indirect target of miR-31 displays reduced levels. β-Actin served as a loading control. D, HEK-293 cells transfected with control antagomiR (black) or anti-miR-31 antagomiR (green) along with reporter constructs containing 3'-UTRs of the predicted miR-31 targets *SRC, RAB27A, NIK*, and *MET. RhoA* 3'UTR reporter construct was used as a positive control. E, same as (D) but with control miR (black), miR-31 (red) and reporter constructs containing 3' UTRs of the predicted miR-31 targets *SRC, RAB27A, NIK, MET*, and *RhoA*.

In order to identify miR-31 target genes in melanoma, we took a multi-pronged approach utilizing, (a) microarray gene expression profiling of Malme3M cells transfected with miR-31, (b) review of full list of miR-31 targets predicted by TargetScan, PITA and miRanda, and (c) melanoma literature search (Supplementary Figure 4). We focused on MET, RAB27a, SRC, and NIK (also known as MAP3K14) as putative target genes as they contain cognate miR-31 binding sites in their 3'-UTR (Figure 4A). Moreover, NIK has recently been reported as a miR-31 target in ATL leukemia [33]. Although the PIK3R3 gene does not harbor a miR-31 binding site in its 3'-UTR, we included it as a potential downstream indirect target of miR-31 since its expression was downregulated in our microarray profile analysis (Supplementary Figure 4C). We first performed immunoblot analysis of all four potential target protein expressions in a panel of nine melanoma cell lines and two normal primary melanocyte culture (Figure 4B). SRC protein was readily detectable in multiple melanoma cell lines and NIK was overexpressed in melanoma cell lines but was detected at low levels in normal melanocytes (Figure 4B). Likewise, MET expression was detected in many of the analyzed cells with the highest expression in SK-Mel5 cells. Interestingly, PIK3R3 was exclusively detected in melanoma cells, with low or undetectable expression in normal melanocytes (Figure 4B). Similarly RAB27a, a gene involved in the regulation of membrane trafficking and exosome formation [37, 38] was highly expressed in a number of melanoma cells.

Three cell lines, MM603, SK-Mel28 and SK-Mel5, were selected for validation since all four miR-31targets SRC, NIK, RAB27a and MET along with PIK3R3 were substantially expressed in these cells. Overexpression of miR-31 by transient transfection in these cells led to significant reduction in SRC, NIK, RAB27a and MET protein levels (Figure 4C). Moreover, PIK3R3, an indirect target of miR-31 also showed reduction in all three cell lines (Figure 4C). Depletion of EZH2 activity by DZNep treatment led to an increase in miR-31 levels (Figure 3C) accompanied by a concomitant reduction in SRC, NIK and RAB27a (Supplementary Figure 5). Reciprocal experiments with miR-31 knockdown were not possible since these cell lines already express very low endogenous miR-31 or in the case of SK-Mel5 cells, miR-31 copies are deleted. To determine whether miR-31 directly targets the 3'UTR of *SRC, RAB27a, NIK* and *MET*, we cloned the full length 3'UTR of these genes into a luciferase reporter. We used 3'UTR of *RhoA* as a positive control since RhoA 3'UTR was reported as a target of miR-31 [30]. Co-transfection of the luciferase reporter 3'UTR plasmids with anti-miR-31 led to significant de-repression of luciferase activity in HEK-293 cells (Figure 4D). Reciprocally, overexpression of miR-31 led to a significant decrease in luciferase activity of these 3'UTR reporters (Figure 4E), demonstrating the inhibitory effect of miR-31 on *SRC, RAB27a, NIK and MET* translation by binding to their 3'UTR.

### miR-31 downregulates EZH2 expression

**Figure 5:**
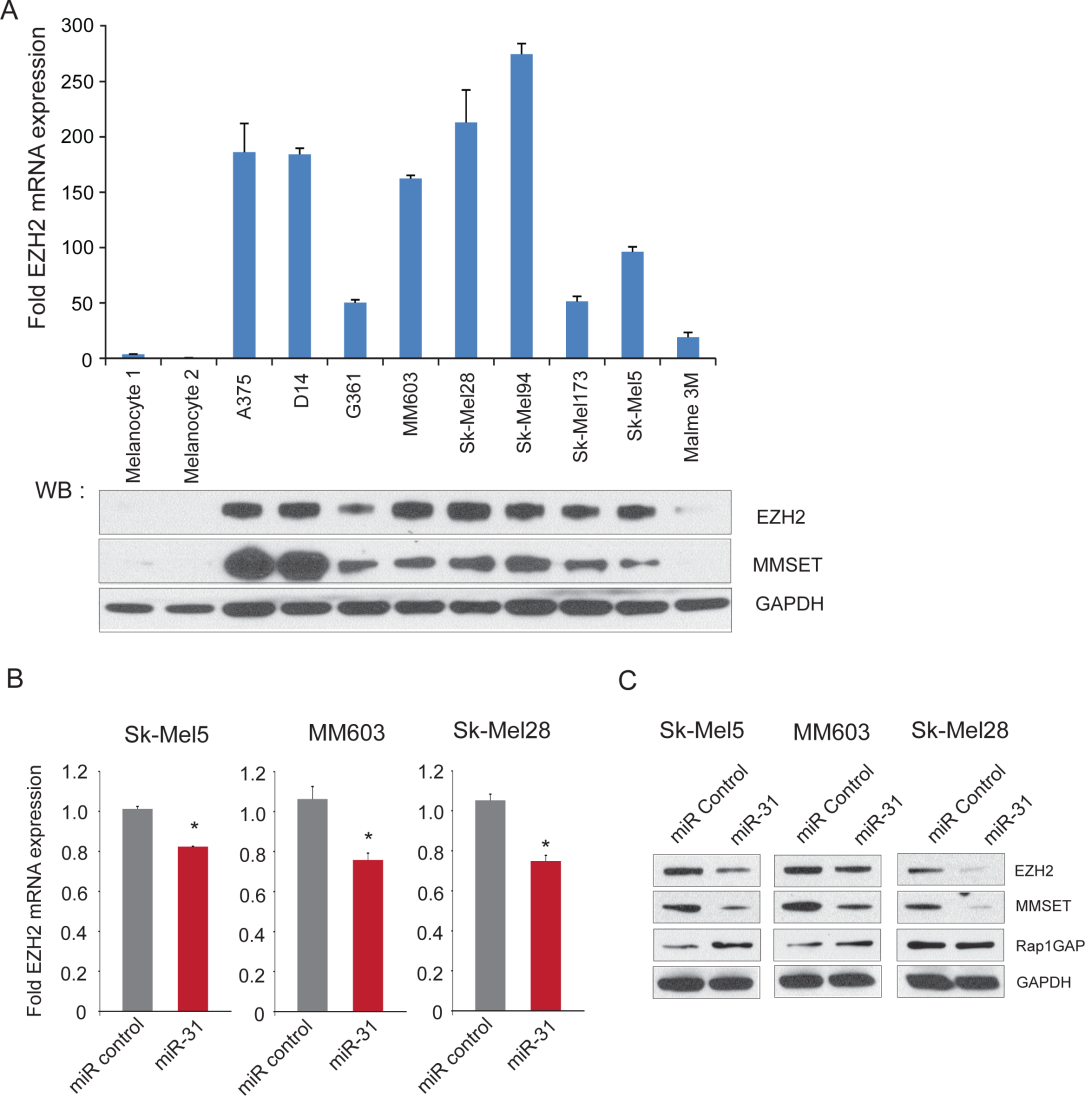
The histone methyltransferase EZH2 is negatively regulated by miR-31 in melanoma A, (*top panel*) qRT-PCR analysis of EZH2 mRNA in melanoma cell lines relative to benign melanocytes. (*bottom panel*) immunoblot analysis of EZH2 and MMSET in benign melanocytes and melanoma cell lines. GAPDH was used as loading control. B, qRT-PCR analysis for EZH2 expression in miR-31 (red) or control miR transfected cells (gray). Expression was normalized with gapdh transcript levels. C, immunoblot analysis for EZH2, MMSET and Rap1GAP using cell lysates from miR-31 and control miR transfected melanoma cell lines. GAPDH served as loading control.

Upon further analysis of Malme3M gene expression profiling data, we noted that EZH2 expression was downregulated by approximately 30% upon miR-31 overexpression (Supplementary Figure 4C). Given that EZH2 acts as a repressor of miR-31 expression, we sought to further characterize the mutually antagonistic regulatory axis between miR-31 and EZH2. We found that high miR-31 expressing primary melanocyte cultures expressed low levels of EZH2 mRNA and protein by qRT-PCR and immunoblotting, respectively. However, melanoma cell lines displayed overexpression of EZH2 mRNA and protein (Figure 5A) and notably the EZH2 mRNA and protein were highly correlated (Spearman r= 0.88, p= 0.0003, Supplementary Figure 6A). The histone methyltransferase MMSET, which is highly correlated with EZH2 in many other solid cancers (Asangani et al., manuscript in review), also demonstrated increased expression in melanoma cell lines relative to primary melanocytes (Figure 5A), and was correlated with EZH2 expression in melanoma cells (Supplementary Figure 6B). Importantly, overexpression of miR-31 resulted in downregulation of EZH2 mRNA and protein (Figures 5B and 5C), and its downstream target MMSET in all three melanoma lines tested. Furthermore, Rap1GAP, known to be repressed by EZH2 [25], was upregulated upon miR-31 overexpression in SK-Mel5 and MM603 cells (Figure 5C). Thus, overexpression of miR-31 resulted in downregulation of EZH2 and subsequently to the modulation of its targets MMSET and Rap1GAP; altered expression of these targets might have contributed to loss of cellular invasion and migration phenotypes as described above (Figure 3).

### Increased expression of EZH2 is associated with melanoma progression and survival

**Figure 6:**
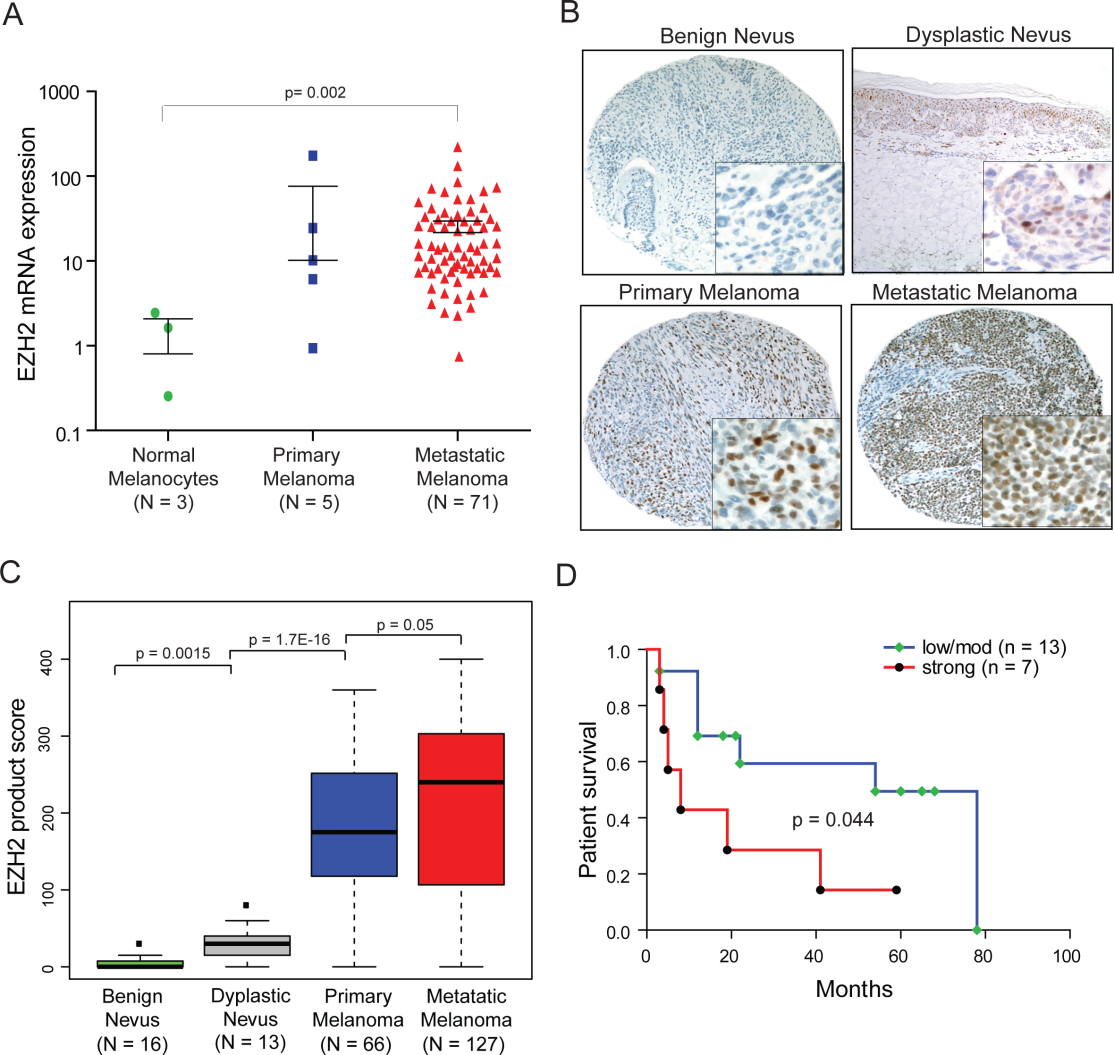
EZH2 is overexpressed in melanoma and correlates with patient survival A, qRT-PCR for EZH2 transcript in a cohort of primary melanoma (blue), metastatic melanoma (red) and benign melanocytes (green). B, representative images depicting immunostaining pattern for EZH2 in benign nevi and melanocytic lesions. Inset magnification 40x showing nuclear positivity for EZH2. C, quantitation of EZH2 immunohistochemical staining, overall staining for EZH2 was measured by multiplying staining percentage (0-100%) by staining intensity on a numerical scale (none=1, weak=2, moderate=3, strong=4), resulting in an overall product score. D, Kaplan-Meier survival analysis showing association between strong EZH2 expression in locoregional metastases and decreased overall survival relative to metastases with moderate/low EZH2 expression (p=0.044, Mantel-Cox log-rank test).

We next measured the expression levels of EZH2 mRNA and protein in a large cohort of melanoma samples. Quantitative RT-PCR analysis demonstrated that EZH2 mRNA was overexpressed in metastatic melanomas (n=71) relative to primary melanocyte cultures (p-value =0.002) (Figure 6A). Primary melanoma tumors (n=5) displayed a trend toward increased EZH2 mRNA expression relative to primary melanocytes that did not reach statistical significance due to small sample size (Figure 6A). EZH2 protein was determined *in situ* by immunohistochemistry utilizing melanoma tissue microarrays (TMA) and individual sections consisting of benign nevus (n=16), dysplastic nevus (n=13), primary melanoma (n=66) and metastatic melanoma (n=127) samples. We found the expression of EZH2 to be low or absent in benign nevi (Figure 6B and C), and EZH2 expression displayed progressive increase from benign nevi, dysplastic nevi, localized and metastatic melanoma samples (p-value =0.0015, =1.7E-16, =0.05 respectively) (Figure 6B and C). To assess whether EZH2 protein expression is associated with overall survival, Kaplan-Meier analysis was carried out for the samples with follow-up data. In univariate Kaplan-Meier analysis, high EZH2 protein expression in locoregional metastases was associated with decreased 5-year survival (p=0.044, Mantel-Cox log-rank test) (Figure 6D). Taken together, these data suggests EZH2 as a highly expressed histone methyltransferase which might be playing a role in melanoma progression leading to poor survival.

## DISCUSSION

In this study we have determined the expression status and regulatory mechanism of miR-31 in melanoma and performed functional characterization of its role in various oncogenic phenotypes and validated novel targets. Although miR-31 has been shown to act as either an oncomiR or tumor suppressor in various tumor types [13], in this study we clearly establish miR-31 as a tumor suppressor, demonstrate that it targets multiple oncogenes such as SRC, MET, NIK and RAB27a and regulates the expression of EZH2 in melanoma.

The gene encoding miR-31 is located within the intron of a long noncoding RNA located on chromosome 9p21. Deletions of chromosome 9p21 are common in melanoma and other tumors and have been shown to include the miR-31 locus in cancers including mesothelioma and urothelial carcinoma [18-20]. By array CGH we observed both heterozygous and homozygous deletions involving the miR-31 locus in 21% of melanomas. The expression of miR-31 was profoundly suppressed in most of the melanoma tissues and cell lines; the loss of expression is attributed to both genomic deletion and epigenetic silencing – a common mechanism of downregulation of tumor suppressors in cancer. Epigenetic modification of chromatin *via* methylation of cytosine residues in CpG islands or by post-translational modification of histones marks transcriptional availability of genes. Polycomb repressor complex 2, of which EZH2 is the catalytic component, mediates trimethylation of histone 3 at lysine K27 (H3K27) and subsequent repression of target genes [24] including miR-31 expression in adult T cell leukemia and prostate cancer [33, 35], and ENCODE ChipSeq data indicates an enrichment of trimethylated H3K27 at the miR-31 promoter region. In addition, miR-31 is silenced in triple-negative breast carcinoma by CpG island methylation [32]. We observed a significant induction of miR-31 expression upon depletion of EZH2 either by RNA interference or by DZNep treatment as well as by treatment with the DNA methylation inhibitor, 5'aza-dC.

The inhibitory effect of miR-31 on cell proliferation has been reported for ATL and mesothelioma [18, 33], however cell growth of only a subset of melanoma cell lines were inhibited upon ectopic overexpression of miR-31 in our studies. Conversely, knockdown of miR-31 led to increased proliferation only in serum-free conditions. miR-31-mediated attenuation of local invasion and metastasis without affecting primary tumor growth was elegantly demonstrated in breast cancer [13, 30], and we observed a similar effect on migration and invasion upon miR-31 overexpression in all melanoma cell lines tested, supporting its role as a metastasis suppressor in melanomas.

In order to identify targets that are regulated by miR-31 to mediate tumor suppressive effects in melanoma, we employed an integrative approach utilizing gene expression microarrays, miR target prediction algorithms, and literature searches. We nominated SRC, MET, NIK and RAB27a as possible miR-31 targets in melanoma; these genes are known to play pro-tumorigenic roles in melanoma [37, 39-42]. The validation of these four melanoma oncogenes as a direct miR-31 target represents a significant finding and elucidates the molecular pathways affected by chromosome 9p21 deletion in melanoma. Moreover, NIK (NF-kB inducing kinase) which promotes cell proliferation and survival in melanoma has recently been validated as a miR-31 target in ATL [33].

**Figure 7:**
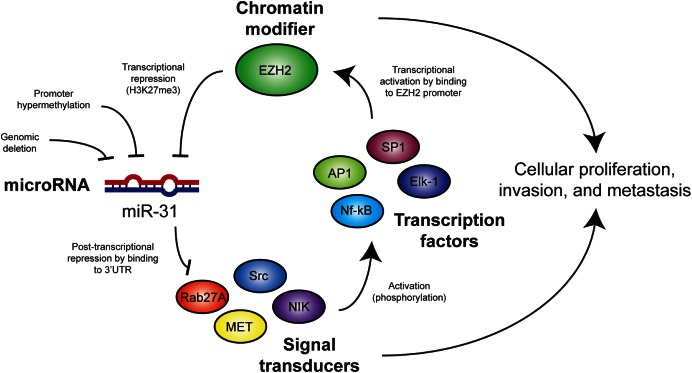
Model for mechanisms of interaction between miR-31 and EZH2 in melanoma cells Targets of miR-31 activate cell signaling pathways that promote EZH2 expression and/or activity. Genomic loss or epigenetic silencing of miR-31 in melanoma leads to increased expression of miR-31 targets and EZH2 activity, which results in further suppression of miR-31 expression and subsequent cancer cell proliferation and metastasis.

Downregulation of EZH2 may represent an additional tumor suppressive role for miR-31 since EZH2 plays a pro-tumorigenic role in melanoma [43]. It is unlikely that EZH2 expression is directly targeted by miR-31 as EZH2 3'UTR lacks a miR-31 binding site. However, EZH2 expression and/or function can be regulated through an alternate mechanism such as MEK-ERK-Elk-1 activation pathway [26, 27]. We observed the presence of multiple cis-regulatory elements for transcriptional factors Elk-1, Sp1, AP1 (c-Jun) and NF-kB in the proximal promoter of EZH2 (Supplementary Figure 7) that are known to be activated by miR-31 targets SRC, MET, and NIK proteins. Therefore, miR-31 may indirectly inhibit EZH2 *via* repression of these upstream activators. Taken together, our findings indicate that EZH2 and miR-31 engage in a mutually antagonistic regulatory circuit that can accelerate tumorigenic progression (Figure 7).

In conclusion, we found recurrent deletion and epigenetic silencing of miR-31 in melanoma. And miR-31 acts as a tumor-suppressive microRNA by exerting inhibitory effects on cell motility and invasion. Importantly, we identified SRC, MET, NIK and RAB27a to be direct targets of miR-31. Moreover, we found that miR-31 also engages in an antagonistic regulatory circuit with EZH2, and loss of miR-31 expression may contribute to EZH2 overexpression in melanoma and provides a potential therapeutic axis which can be targeted by small molecule inhibitor.

## METHODS

### Cell lines

If not indicated otherwise, melanoma cell lines were obtained from the American Type Culture Collection (ATCC) and cultured in DMEM with 10% FBS. SK-Mel5, SK-Mel28, and MM603 melanoma cell lines were provided by M. Soengas, and cultured in DMEM with 10% FBS. Primary foreskin melanocyte cultures were provided by M. Verahaegen and cultured in Media 254CF (Cascade Biologics) with human melanocyte growth supplement (S-002-5, Cascade Biologics).

### Tumor samples

All human subjects studies were approved by the Institutional Review Board of the University of Michigan. All tumor tissue was procured from patients who underwent excision at the University of Michigan Health System between the years of 2007 and 2010. At time of collection, tumor tissue was flash-frozen in liquid nitrogen and stored at −80^°^C until RNA or DNA extraction. Formalin-fixed paraffin-embedded tissue for tissue microarray construction was obtained from archival tissue blocks. Human epidermal melanocyte (HEM) RNA was obtained from Cell Applications, or collected from cultured primary melanocytes.

### array–Comparative Genomic Hybridization (aCGH)

aCGH of tumor and cell line samples was performed using gDNA on Agilent's 244K aCGH microarrays (Human Genome CGH 244K Oligo Microarray) as previously described [44].

### RNA and DNA isolation

For patient tumor samples, areas containing at least 70% tumor were targeted for RNA isolation using hematoxylin and eosin (H&E) stains obtained on frozen sections for each specimen. Representative areas were removed from the tissue block and homogenized in the presence of Trizol reagent (Life Technologies) and total cellular RNA was purified according to the manufacturer's standard protocol. For cell lines, total RNA was isolated using RNeasy Mini Kit (Qiagen) according to the manufacturer's protocol. After purification, RNA quality was assessed by Agilent Bioanalyzer. DNA was isolated using the DNeasy Blood & Tissue kit (Qiagen).

### Quantitative real-time PCR

All PCR reactions were performed on StepOne real-time PCR system (Applied Biosystems) using SYBR Green or Taqman detection. For analysis of relative genomic copy number, primers for pri-miR-31 (Applied Biosystems assay ID Hs03302684_pri) or the reference gene PGK1 (Roche 5046173001) were utilized in reactions with 50ng genomic DNA. Stem-loop RT-PCR analysis of miR-31 expression was performed using the hsa-miR31 microRNA Assay (Applied Biosystems assay ID 002279) as described previously [45]. RNU6B expression for normalization was prepared and analyzed using the RNU6B microRNA Assay (Applied Biosystems assay ID 001093). Results were analyzed using the 2^−^*DDCT*-method. SYBR Green PCR primers used in this study are listed in *Table S1*.

### RNA interference and microRNA transfections

Knockdown of EZH2 was accomplished by RNA interference using two different commercially available and well-characterized siRNA duplexes (Dharmacon, Lafayette, CO). Precursors of miR-31 (Ambion PM11465), negative control miR (Ambion 17110), anti-miR-31 (Ambion 11465) and negative control anti-miR (Ambion 17010) were purchased from Ambion (Austin, TX). Transfections were performed with OptiMEM (Invitrogen) and oligofectamine (Invitrogen) as previously described [28].

### Antibodies and Immunoblot analyses

Antibodies used in the study are listed in *Table S2* and were employed at dilutions recommended by the manufacturers. For miR-31 overexpression studies, cell lines SK-Mel5 (having miR-31 genomic deletion), SK-Mel28, and MM603 were transfected with miR-31 as described above, and protein was extracted 72 hours post-transfection. Cells were homogenized in RIPA buffer containing complete proteinase inhibitor (Roche, Indianapolis, IN, USA), and protein concentrations were determined by BCA kit. For immunoblot analysis, 200ug total protein extract was boiled in sample buffer and 10-15ug aliquots were separated by SDS-PAGE and transferred onto Polyvinylidene Difluoride membrane (GE Healthcare). The membrane was incubated for half an hour in blocking buffer [Tris-buffered saline, 0.1% Tween (TBS-T), 5% nonfat dry milk] followed by incubation overnight at 4°C with the primary antibody. Following a wash with TBS-T, the blot was incubated with horseradish peroxidase-conjugated secondary antibody and signals were visualized by enhanced chemiluminescence system as per manufacturer's protocol (GE Healthcare).

### Cell Proliferation assay

Cells were transfected with precursor miR-31 or negative miR control. After 24 hours, cells were plated into 6-well plates at a density of 2×10^4^ cells/well. Cells were trypsinized and cell counts were analyzed on a Coulter counter (Beckman Coulter, Fullerton, CA) at indicated time points.

### Motility and basement membrane invasion assay

Cells were transfected with precursor miR31 or negative control miR. After 24 hours, cells were seeded on 8 μm inserts (BD Falcon) coated with Matrigel (for invasion assays) or uncoated (for motility assays) in a 24-well culture plates. Fetal bovine serum was added to the lower chamber as a chemoattractant. After 72 hours non-invading cells were gently removed by cotton swab. Invasive/migrated cells on the lower side of the chamber were stained with crystal violet and air-dried. Relative invasion/migration was quantitated by solubilization of crystal violet dye and measurement of absorbance at 560 nm.

### 5' aza-deoxycytidine and DZNep treatment

Cells were seeded in 6-well plates and treated the following day with the indicated concentrations of 5-aza-2'-deoxycytidine (EMD Biosource) or an equivalent volume of DMSO vehicle. 5' azacytidine was renewed daily for five days of treatment. Similarly, DZNep was added to the cells and cultured for four days. Total RNA and protein were extracted for PCR or immunoblot analysis.

### Construction of 3'UTR-luciferase plasmids and reporter assays

The SRC 2.0kb full length 3' UTR Luciferase reporter plasmid was purchased from GeneCopoeia (Rockville, MD). The full length 3'UTR of NIK, MET, RAB27a and RhoA was PCR amplified using cDNA and inserted into Topo-TA cloning vector (Invitrogen), and after sequence confirmation subcloned into pEZX-MT01 vector (GeneCopoeia). For 3'UTR luciferase reporter assays, HEK-293 cells were cultured in 24-well plates and co-transfected with the 1μg reporter construct and 50nM precursor miR or negative control miR in duplicates or triplicates using FuGENE^®^ HD (Promega). 48 hours post-transfection luciferase activities were measured using Luc-Pair miR Luciferase Assay kit (GeneCopoeia, Rockville, MD) and normalized by Renilla luciferase activity from matched wells.

### Immunohistochemistry

For immunohistochemical analysis three tissue microarrays (TMA) were used, containing a total of 4 benign nevi, 1 dysplastic nevus, 63 primary melanomas, and 127 metastatic melanomas, with each case represented by triplicate 0.6 mm cores. Immunohistochemistry was also performed on individual slides for 12 benign nevi, 12 dysplastic nevi, and 4 primary melanomas. Tumor content of each core or slide was verified by H&E stain. Immunohistochemistry was performed as previously described [22], using anti-EZH2 antibody at 1:100 dilution. Slides were scored independently by two pathologists (PH and LK) for percentage of positive cells and intensity of staining. All positive cases displayed nuclear staining. Photomicrographs were taken with a SPOT Insight Color camera (Diagnostic Instruments) on an Olympus BX41 microscope with Olympus UPlanFL 10x and 40x objectives using SPOT Basic software.

### Statistical Analysis

All statistical analysis was carried out using GraphPad Prism software program. For quantitative data, treatment groups were reported as mean ± SEM and compared using the unpaired Student's t-test. For Kaplan-Meir survival analysis, EZH2 expression values were categorized into low/moderate (<300 product score) and high (>300 product score). Statistical significance was established at p ≤ 0.05 unless otherwise noted.

## Supplementary Figures and Tables




