# Supplementary material for: Genetic and epigenetic loss of microRNA-31 leads to feed-forward expression of EZH2 in melanoma
Source: Oncotarget. 2012 Aug 31;3(9):1011–25. doi: 10.18632/oncotarget.622 (PMC3663613; doi:10.18632/oncotarget.622)
Supplement: Supplementary file 1 [file oncotarget-03-1011-s001.pdf]

Genetic and epigenetic loss of microRNA-31 leads to feed-forward expression of EZH2 in melanoma – Asangani et al

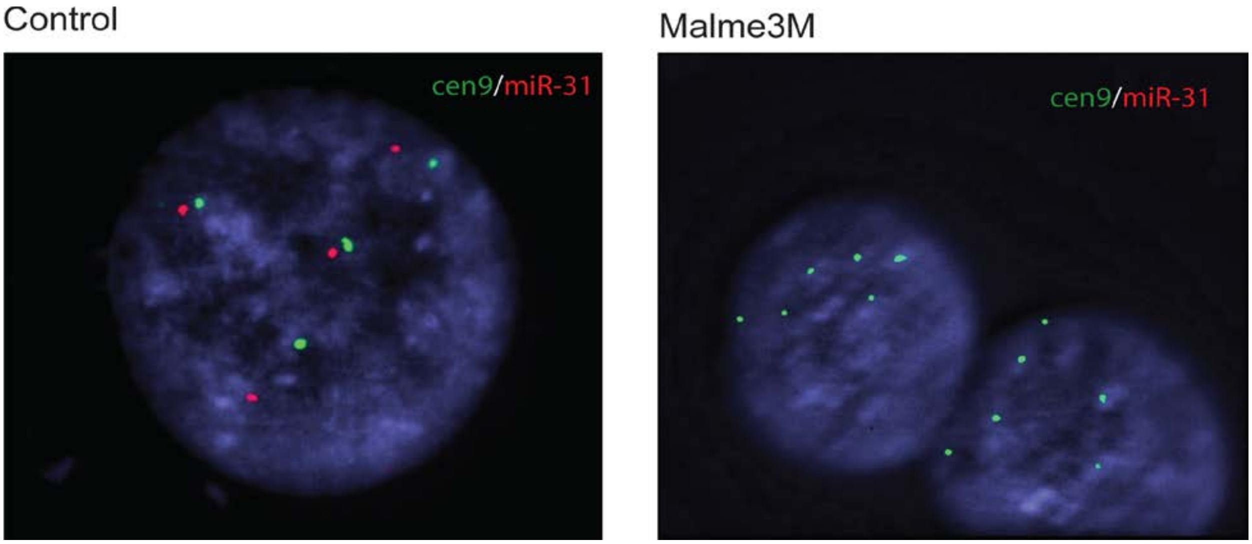

Figure S1: By fluorescence in-situ hybridization, Malme-3M melanoma cells display homozygous loss of the hsa-miR-31 locus (red). Chromosome 9 centromere (green probe) is included as a control.

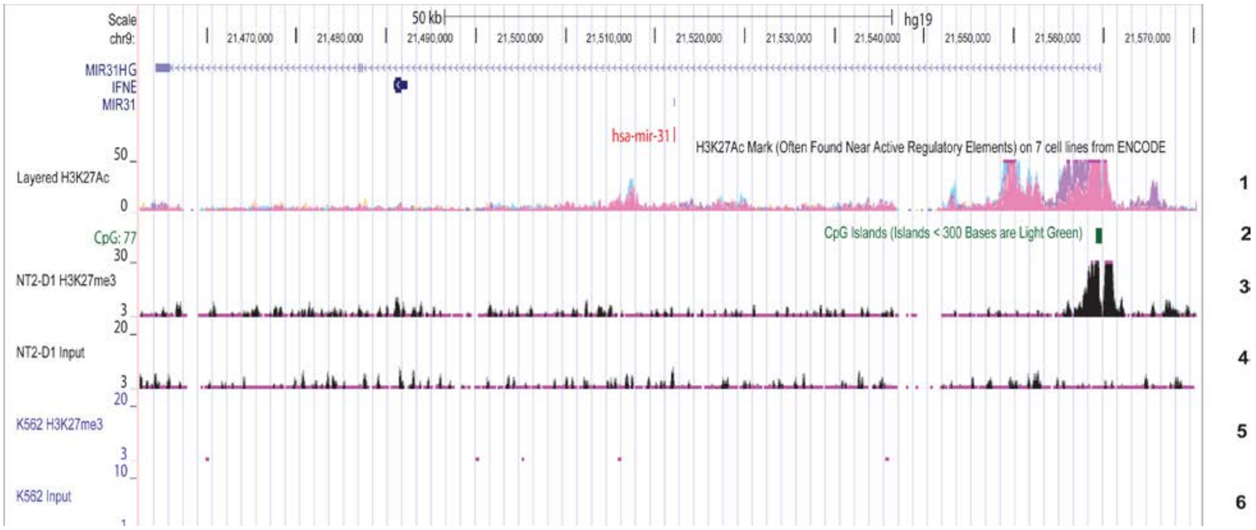

Figure S2: UCSC Genome browser view of miR-31 loci in the human genome. Mature miR-31 is encoded in the intron of miR31HG (miR-31 host gene) long non-coding RNA consisting of 4 exons on Chr 9p21. Regulatory region of miR31HG shows enrichment for H3K27Ac mark associated with transcriptionally poised genes, in 7 cell lines ChIP-seq data from encode, indicated with ←1. The same regulatory region near the transcription start site consists of 77

CpG islands validated for methylation in multiple cell lines (encode data not shown here). The CpG island track is indicated with ← 2. ChIP-seq enrichment for EZH2 associated transcriptional silencing H3K27me3 mark on the regulatory region is seen for NT2-D1 ChIP-seq data, indicated with ← 3. The input ChIP-seq tracks is indicated with ← 4 showing no enrichment peaks. The K562 ChIP-seq for H3K27me3 or input does not show any enrihment for this region due to the focal deletion of miR-31 region in this cell line.

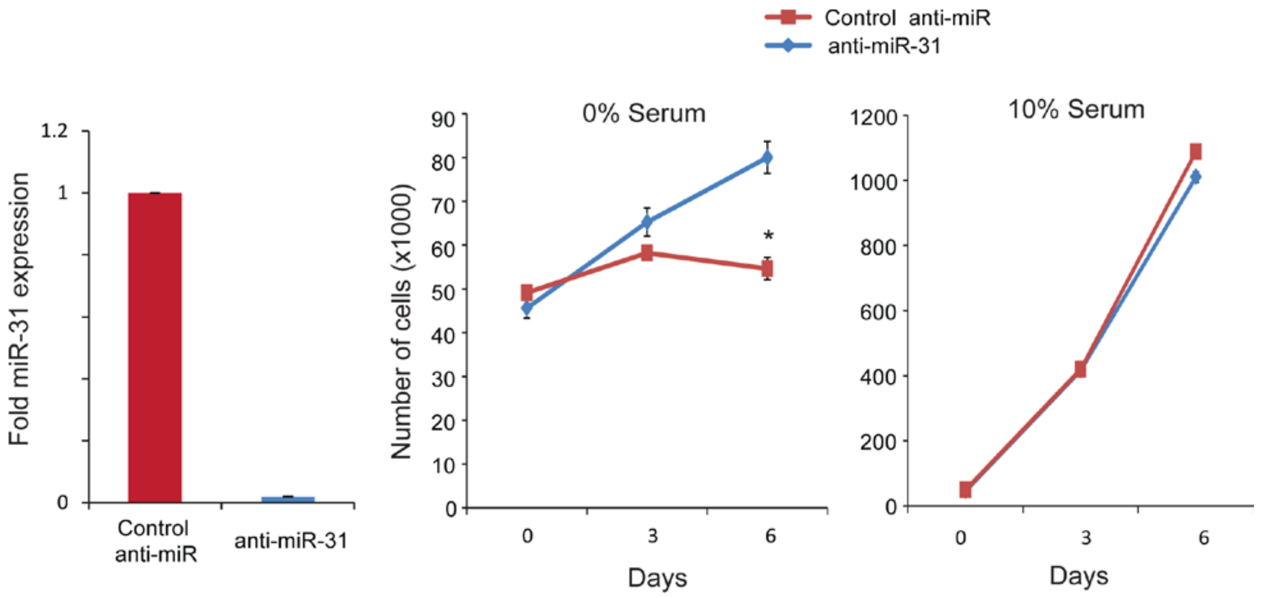

Figure S3: Inhibition of miR-31 promotes growth of A375 melanoma cells in serum-free conditions. A375 cells were transfected with anti-miR-31 or anti-miR control. qRT-PCR for miR-31 and U6 snRNA using total RNA was determined as described in methods section. Cell number was quantitated by Coulter counter at the indicated time points.

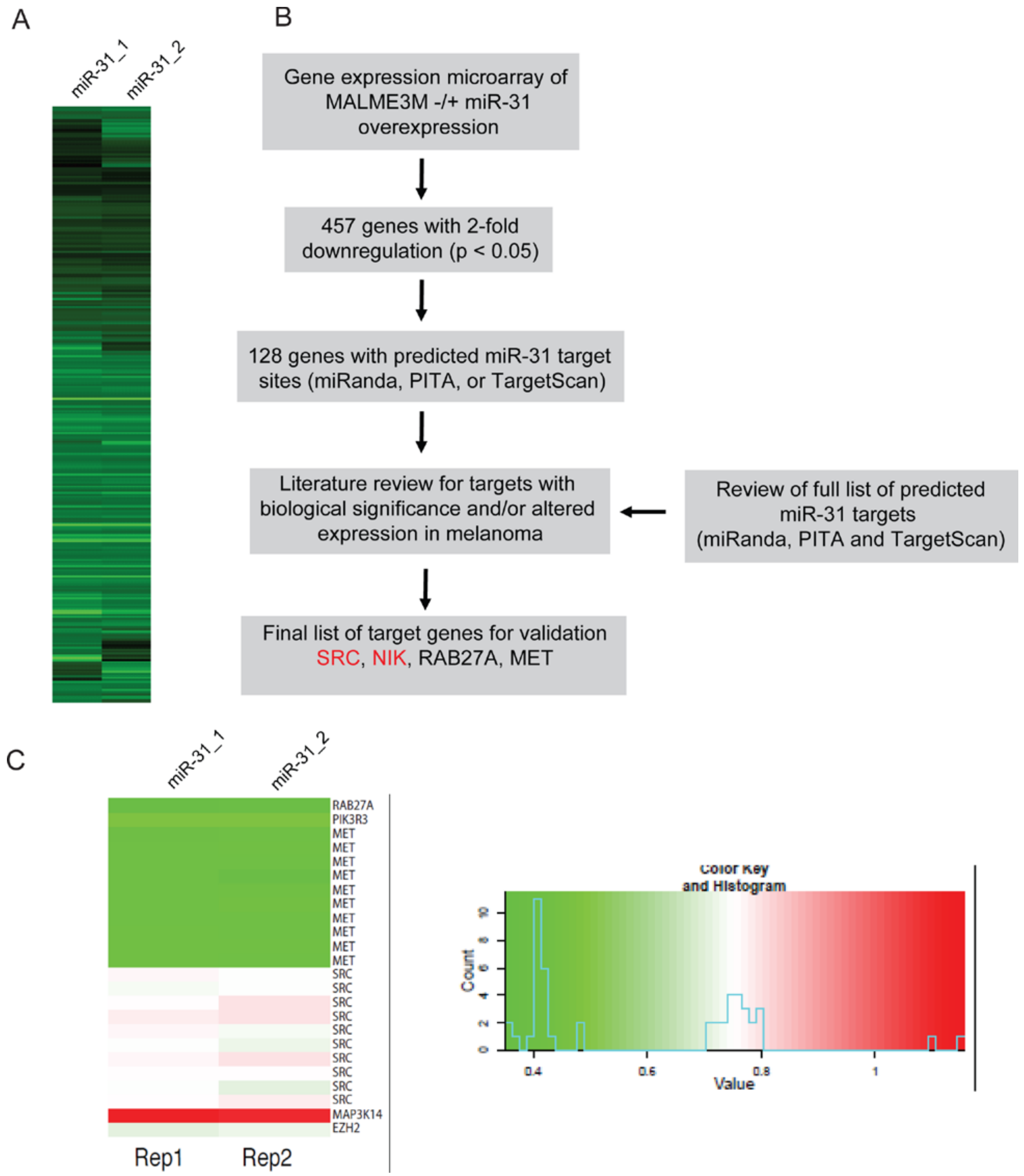

Figure S4: Identification of miR-31 targets by expression microarray and target prediction algorithms. A, heat map of genes downregulated in Malme3M melanoma cells upon miR-31 overexpression, as determined by gene expression microarray. Results of duplicate microarrays are shown. B, strategy for miR-31 target identification. Final targets highlighted in red displayed downregulation at the protein level, but not the mRNA level. C, heat map showing potential miR-31 targets MET, RAB27A, SRC, and NIK along with EZH2 expression in miR-31 overexpression microarray profiling data.

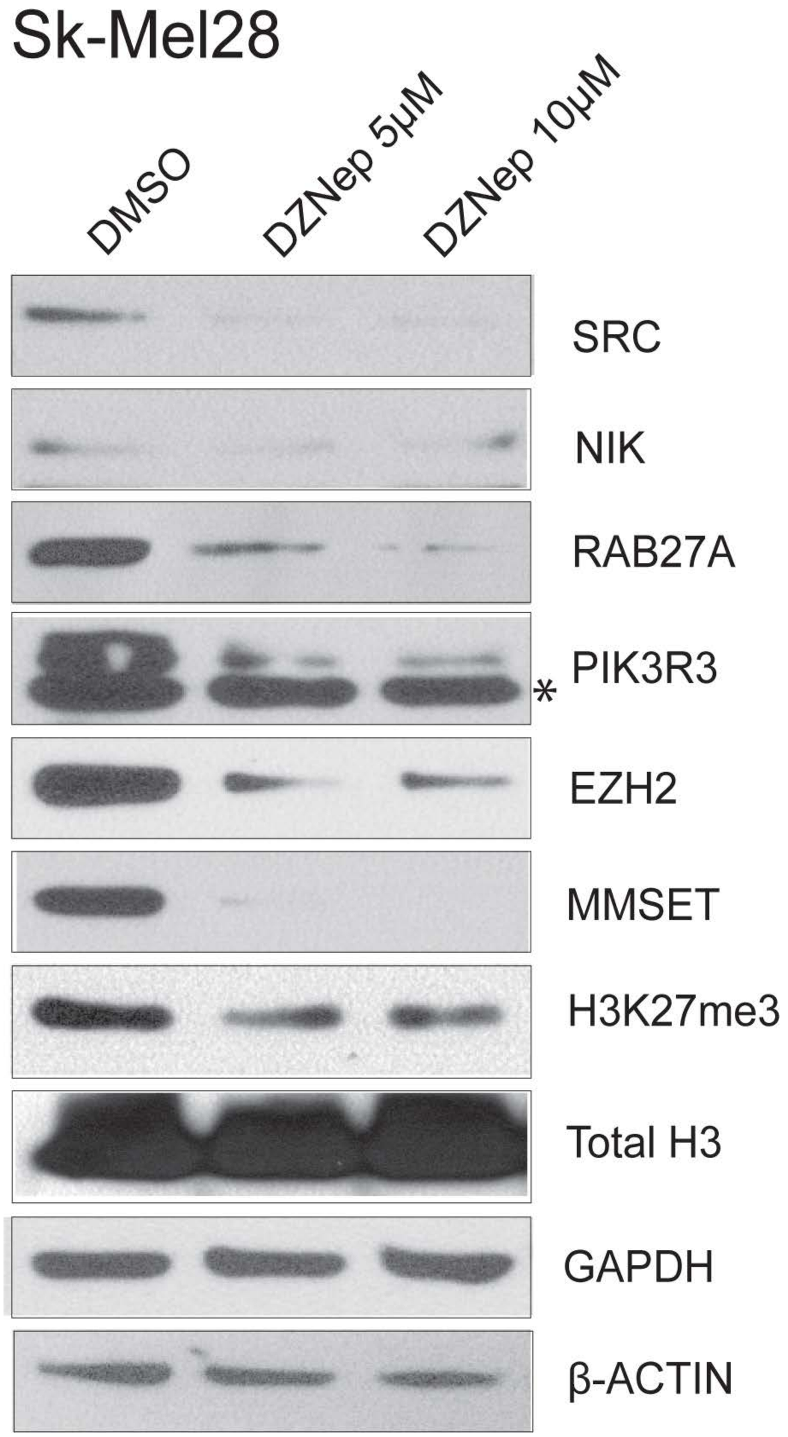

Figure S5: DZNep treatment results in decreased expression of miR-31 targets. Sk-Mel28 cells were treated with DZNep for 4 days and the levels of EZH2, H3K27me3, MMSET, along with miR-31 targets SRC, NIK, RAB27a and PIK3R3 were examined by immunoblotting using total cell extracts. Total H3, GAPDH and β-Actin were used as a loading control.

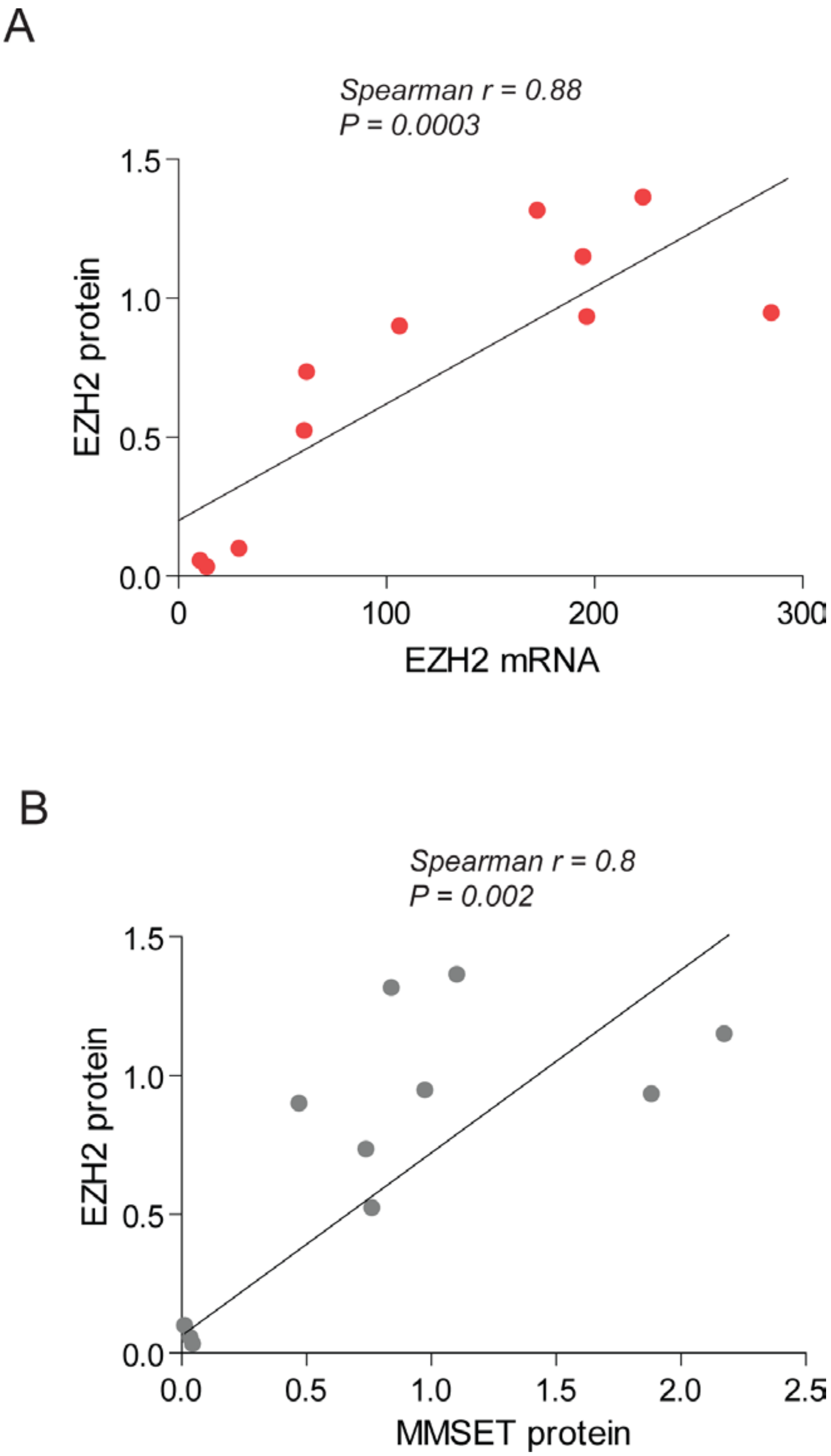

Figure S6: EZH2 mRNA and protein expression is highly correlated. A, graph showing correlation between EZH2 mRNA and protein in a panel consists of benign melanocytes and melanoma cell lines. For EZH2 protein expression values, immunoblot band intensities from Figure 5A were measured using ImageJ and normalized against β-Actin. **B**, graph showing correlation between EZH2 and MMSET proteins in the same cell lines.

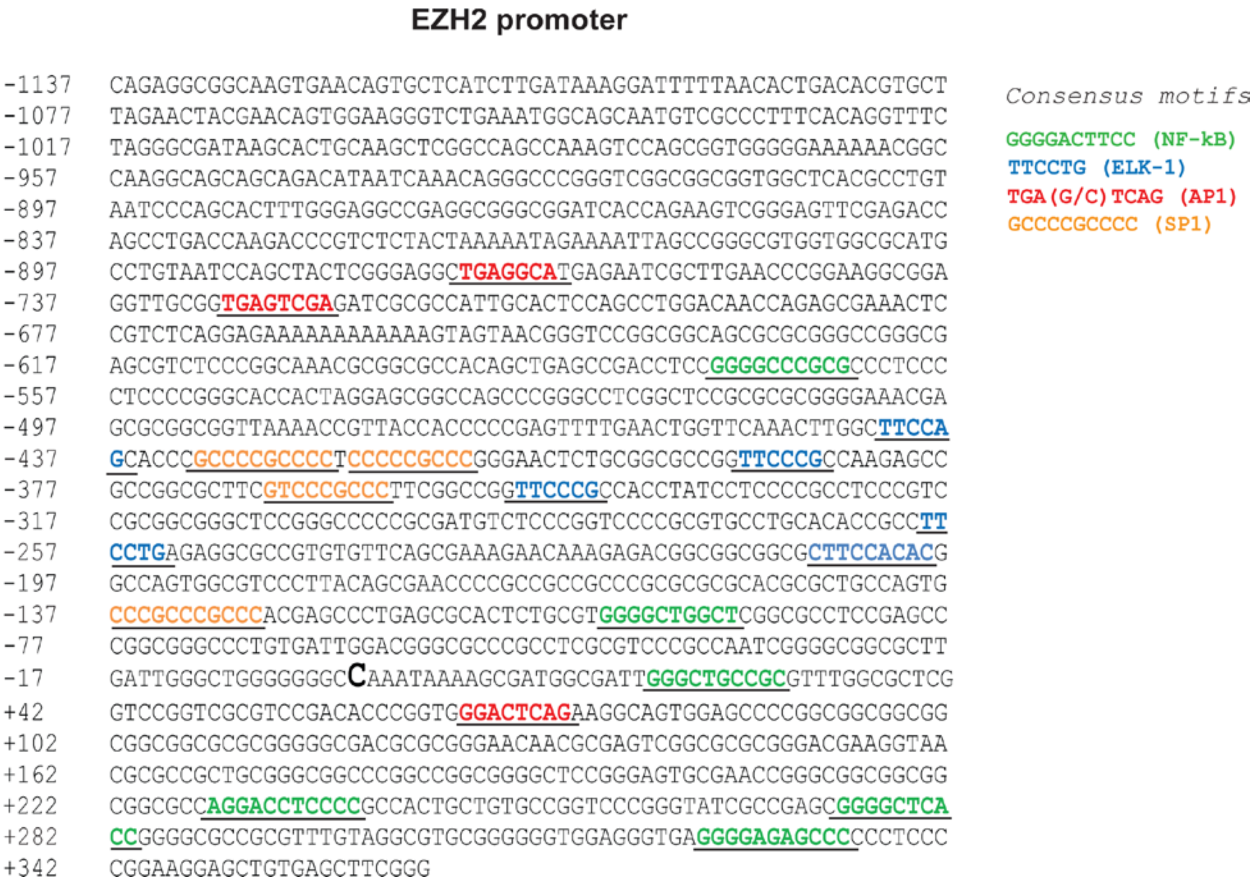

Figure S7: EZH2 promoter. Nucleotide sequence of the 5'- flanking region of the EZH2 gene. Transcription start site +1 is indicated with bold letter, underlined colored sequences represent transcription factor binding sites. Inset shows consensus motif sequence for the given transcription factor.

Supplemental materials and methods

Fluorescence in-situ hybridization

Interphase fluorescence in-situ hybridization (FISH) was performed on MALME-3M and MM576 (control) melanoma cells as described (1). Slides were examined using the ImagingZ1 microscope (Carl Zeiss). FISH signals were scored manually (100x oil immersion) in morphologically intact and nonoverlapping nuclei. Fluorescence images were captured using a high resolution CCD camera controlled by ISIS image processing software (Metasystems, Germany).

Cell growth in serum-free conditions

Transfection of A375 melanoma cells with anti-miR-31 or negative control anti-miR was performed with OptiMEM (Invitrogen) and oligofectamine (Invitrogen) as previously described ((2). Two serial transfections were performed in order to maximize knockdown. After 24 hours, cells were plated into 6-well plates at a density of $2x10^4$ cells/well. After cells had become fully

adherent, cells were washed twice with PBS and cultured in serum-free media. Cell counts were estimated by trypsinizing the cells and analysis was done using a Coulter counter (Beckman Coulter, Fullerton, CA) at the indicated time points.

Gene expression profiling

RNA extracted from Malme-3M melanoma cells transfected with pre-mir-31 or control precursor miR was subjected to expression profiling using the Agilent Whole Human Genome Oligo Microarray (Santa Clara, CA) as previously described (3). Significant downregulation was defined as at least a 2-fold decrease in expression with p-value ≤ 0.05 in both the replicates.

Supplemental references

1. Palanisamy N, Ateeq B, Kalyana-Sundaram S, Pflueger D, Ramnarayanan K, Shankar S, et al. Rearrangements of the RAF kinase pathway in prostate cancer, gastric cancer and melanoma. Nat Med.16:793-8.
2. Varambally S, Cao Q, Mani RS, Shankar S, Wang X, Ateeq B, et al. Genomic loss of microRNA-101 leads to overexpression of histone methyltransferase EZH2 in cancer. Science. 2008;322:1695-9.
3. Cao Q, Mani RS, Ateeq B, Dhanasekaran SM, Asangani IA, Prensner JR, et al. Coordinated regulation of polycomb group complexes through microRNAs in cancer. Cancer Cell. 2011;20:187-99.
