# Supplementary material for: Genetic and epigenetic loss of microRNA-31 leads to feed-forward expression of EZH2 in melanoma
Source: Oncotarget. 2012 Aug 31;3(9):1011–25. doi: 10.18632/oncotarget.622 (PMC3663613; doi:10.18632/oncotarget.622)
Supplement: Supplementary file 2 [file oncotarget-03-1011-s002.docx]

Genetic and epigenetic loss of microRNA-31 leads to feed-forward expression of EZH2 in melanoma – Asangani et al
